# Diversity of Water Frogs *Pelophylax* spp. in Turkey: Do Mating Vocalizations Mirror Nominal Taxon Delimitation? †

**DOI:** 10.3390/ani13111725

**Published:** 2023-05-23

**Authors:** Ulrich Sinsch, Stefan Werding, Uğur Kaya

**Affiliations:** 1 Institute of Integrated Sciences, Department of Biology, University of Koblenz, D-56070 Koblenz, Germany; stefan_werding@gmx.de; 2 Biology Section, Zoology Department, Faculty of Science, Ege University, Bornova-İzmir 35100, Turkey; ugur.kaya@ege.edu.tr

**Keywords:** advertisement call, release call, female call, *Pelophylax ridibundus*, *Pelophylax bedriagae*, *Pelophylax caralitanus*

## Abstract

**Simple Summary:**

National conservation management is informed by knowledge on the species diversity of the taxon group in focus, specifically on the occurrence of endemics. Turkey, at the intersection of the Caucasus, Irano-Anatolian, and Mediterranean biodiversity hotspots, harbors a rich anuran diversity including two species considered endemic. The species status of one of them, the Beyşehir water frog *Pelophylax caralitanus*, has been in question since its proposal in 2001. We provide evidence that mating vocalizations of this taxon do not differ sufficiently from those of the widespread *P. bedriagae* to function as a premating barrier between the two, preventing significant gene flux. The low degree of bioacoustic differentiation agrees with earlier genetic, karyological, and morphological findings. We conclude that the Beyşehir frogs do not deserve species status and that *P. caralitanus* are in fact *P. bedriagae*. Therefore, the number of endemic anuran species in Turkey decreases to one.

**Abstract:**

Informed conservation management requires exact knowledge on the species diversity of the taxon group in focus within a geographic area, specifically on the occurrence of endemics. In Turkey, there are three water frog taxa of the genus *Pelophylax*; one is the widespread *P. bedriagae*, the other two are geographically restricted to either Thrace (*P. ridibundus*) or to the Anatolian Lake district (*P. caralitanus*). The species status of the Beyşehir frog *P. caralitanus* has been questioned since its proposal in 2001. We recorded and analyzed advertisement and release vocalizations at representative populations of *Pelophylax* taxa to assess the degree of inter-taxon differentiation and the potential for premating isolation. We found that *P. bedriagae* and *P. caralitanus* have much more similar vocalizations than both have compared to *P. ridibundus*. A functional bioacoustic premating barrier between *bedriagae* and *caralitanus* in syntopy does not exist according to our study. The low degree of bioacoustic differentiation mirrors earlier genetic, karyological, and morphological findings. We conclude that the Beyşehir frogs do not deserve species status and that *P. caralitanus* should be considered a junior synonym of *P. bedriagae*. Therefore, the number of endemic anuran species in Turkey decreases to one.

## 1. Introduction

In the era of human-mediated climate change, informed conservation management requires reliable knowledge on the actual species diversity in a region or at national scale [1]. Vertebrate diversity is a major focus of conservation management and comparatively well-known in Turkey, which marks an area of exchange between European and Asian faunal elements [2]. The identification of key biodiversity areas in Turkey yielded 313 hotspots from which 2/3 are threatened by irrigation, drainage, and dam projects [3]. This is alarming, specifically with respect to Turkish Amphibia that are species-rich and particularly affected due to their dependence on moist habitats [4]. For example, the Anatolian and the Western Aegean regions still hold a high number of amphibian species [5,6]. Unsustainable harvest and Bd infections threaten many populations of water frogs *Pelophylax* spp. [7,8]. Even common species such as the Levantine frog *P. bedriagae* are currently suffering declines [9].

The diversity of *Pelophylax* frogs has long been underestimated in Turkey, with early reviews considering them a single species, *P. ridibundus* (Pallas, 1771) [10,11]. This view was challenged in 1992, when Schneider et al. showed that most of Turkey was inhabited by a second species, now identified as *P. bedriagae* (Camerano, 1882), whereas *P. ridibundus* was restricted to Turkish Thrace [12,13,14]. These two species show postzygotic reproductive isolation, significant differences in advertisement call structure, and DNA sequence differences at the species level (3.1% uncorrected P-distance) [15,16]. Presently, a third species, *P. caralitanus,* has been listed for the southwestern Anatolian Lake district, considered one of the two endemic anurans of Turkey [17]. However, the taxonomic status of these water frogs has remained in discussion since the seminal study of Bodenheimer, who noticed that water frogs from Lake Beyşehir differed from others by their large size and the orange-colored venter [10]. Some authors consider them conspecific with *P. bedriagae* [14,18], others a subspecies of *P. bedriagae* [16,19,20,21,22,23,24,25,26,27], and few a species distinct from *P. bedriagae* [28,29]. The most recent molecular study raises substantial doubts on the proposed species status because sequence differences at Cyt b, 12S rRNA, and 16S rRNA loci yielded low genetic distances of 0.6–1.2% within the *P. bedriagae/caralitanus* clade and mtDNA haplotypes of *P. caralitanus* and the presence of orange belly coloration did not concur [16,26]. Karyological evidence suggests that the “Lake district taxon” (=*P. caralitanus*) includes hybrid lines with coastal populations (=*P. bedriagae*) [30]. These findings contrast with the species claim of Jdeidi et al. [28,31]. The claim is essentially based on a flawed discriminant analysis of a morphometric data set (absence of control for size effects) and the falsified assumption that the orange belly is an apomorphic feature of *P. caralitanus*. Moreover, Jdeidi et al. suggest in a congress abstract that advertisement calls are distinctive from *P. bedriagae* without presenting supporting data [31]. Consequently, a novel approach is needed to answer the question if two or three species are involved in the water frog diversity of Turkey.

Our novel approach includes (1) holistic integration of the information coded in all types of vocalizations emitted during mating, i.e., advertisement and release calls, and (2) adjustment of all call parameters prior to multivariate analyses to eliminate potential effects of ambient temperature and body size on call structure. We focus on the acoustic communication of water frogs from representative populations of each taxon during mating, i.e., on the potential of premating isolation [32,33,34]. Mating vocalizations serve, on one hand, to attract conspecific females to advertising males and, on the other, to avoid male–male amplexus and unwanted clasping of females by emitting release calls. The two types of mating vocalizations constitute potential barriers for mismating among water frogs of different taxa. Consequently, the distinctiveness of *P. ridibundus* and *P. bedriagae* calls serves as a baseline for the study of the *P. bedriagae*/*P. caralitanus* system. Analogous to the situation in the contact zone between *P. ridibundus* and *P. kurtmuelleri* in Greece, we predict that advertisement calls of *P. bedriagae*/*P. caralitanus,* which co-occur in the surroundings of the Lake district, differ from each other at the level of those of *P. bedriagae* and *P. ridibundus*, maybe stronger due to character displacement if they are distinct species [35]. The rationale is that two closely related taxa are expected to have bioacoustic premating barriers in sympatry to prevent interspecific gene flux. In contrast, we predict that release calls given by males and females to avoid mismatings are more similar in structure than the advertisement calls are. The aim of this study is to provide a scientifically based evaluation, for conservation issues, as to whether *P. caralitanus* is an endemic frog species of Turkey.

## 2. Materials and Methods

### 2.1. Study Populations and Sampling

Water frog populations representative for the three nominal species of the genus *Pelophylax* in Turkey have been sampled in 2002 at four sites: (1) *P. ridibundus* in Turkish Thrace, Edirne (41°40′2″ N, 26°30′14″ E, 42 m a.s.l.); (2) *P. bedriagae* in Izmir (38°24′32″ N, 27°3′8″ E, 2 m a.s.l.); (3) *P. caralitanus* at the shores of Lake Beyşehir, Konya province (type locality, 37°40′0″ N, 31°42′40″ E, 1100 m a.s.l.); and Lake Eber, Afyon Province (38°36′54″ N, 31°8′11″ E, 967 m a.s.l.). Voucher specimens were captured at these sites by U.K. and transported to the laboratory in Koblenz, Germany, for further investigations (exportation permit: General Directorate of Protection and Control, Ministery of Agriculture and Rural Affairs, ref. 242, 18 August 2002). We collected 13 *P. ridibundus* (2 males, 11 females) with a snout-vent length (distance between the tip of snout and the posterior margin of urostyle) ranging from 66.5 to 84.1 mm, 12 *P. bedriagae* (5 males, 7 females) with an SVL range of 59.8 mm to 88.9 mm, and 24 *P. caralitanus* (6 males, 18 females) with an SVL range of 62.8 mm to 121.3 mm. Following call records and extraction of DNA material, the specimens were euthanized using an overdose of MS 222, preserved in 10% formaldehyde, and integrated into the zoological collection of the University of Koblenz (collection numbers: UniKoZC2002.05-2002.54).

We adhered to the ASAB/ABS Guidelines for the Use of Animals in Research. The animal study protocol was approved by the Ethics Committee of Fachbereich 3, Department of Biology, Zoology Group, University of Koblenz, Germany (Si3/2002/1, date of approval: 14 July 2002) and by the Faculty of Sciences, Department Biology, Section of Zoology, Ege University (date of approval: 29 July 2002).

### 2.2. Call Records and Analyses

In the natural environment, spontaneously given advertisement and territorial calls of focal individuals were recorded with a condenser microphone (Sennheiser, Wedemark, Germany, Type MKH 415 T) and a portable tape recorder (Uher, Bad Homburg, Germany, 4000 S) and the corresponding water temperature with a digital thermometer (Technoterm 1500). No effort was made to collect the recorded specimens. Therefore, SVL of the calling males was not available. In the laboratory, vocalizations evoked in response to an artificial amplexus were recorded at 20–22 °C air temperature for about 1 min per individual using a microphone (Sennheiser, Wedemark, Germany, Type MKH 415 T) and a DAT recorder (Sony Deutschland GmbH, Cologne, Germany, TCD-D100). The artificial amplexus consisted of gently compressing the sides of the frog held between thumb and forefinger at 10 cm from the frog’s snout [36,37,38]. For quantitative call descriptions, we analyzed and visualized the spectral and the temporal structure of vocalizations using ADOBE Audition 1.0. Stereo recordings were converted to mono using a sampling rate of 44.1 kHz and resolution of 16 bits. Audio spectrograms and frequency analyses were prepared by applying Blackman–Harris Fast Fourier transformation with an FFT width of 1024 points.

Advertisement calls were characterized by eight variables (definitions according to [14,39]: (1) call duration [ms]; (2) number of pulse groups (=notes) per call [N]; (3) pulse group duration [ms]; (4) inter-pulse group interval [ms]; (5) pulse group repetition rate [N/s]; (6) pulses per pulse group [N]; (7) pulse repetition rate within a pulse group [N/s]; (8) dominant frequency of complete call [Hz]. Depending on the structure of the evoked call’s release recorded during artificial amplexus, we used a subset of the advertisement call parameters for description.

### 2.3. Statistical Procedures

Descriptive statistics of call parameters refer to the raw data set and include mean and standard error. For statistical comparison among taxa, data were log10-normalized because some variables distributions deviated from normality. We used Analyses of Co-Variance (ANCOVA, type III sums of squares) with species as fixed factors and water temperature (advertisement calls) or SVL (release/female calls) as continuous co-variables. As the calls evoked by artificial amplexus were recorded at the same air temperature, temperature was not included as a co-variable. In turn, SVL was unknown for the advertising males, and, therefore, call structure could not be adjusted for size of the caller. For multiple group comparisons, we used a multiple range test with Bonferroni correction. To obtain a quantitative measure for the dissimilarity of call structure in the three taxa, we applied discriminant analyses with a priori defined groups (=taxa). The basic advertisement call data set included eight parameters (averages of 3–10 calls of a series) and the corresponding water temperature with n observations (=individuals). As several parameters are correlated with temperature, we calculated linear regression models for each parameter and stored the corresponding studentized residuals as temperature-independent derived data. The residual matrix was used as an input data set for the discriminant analysis. Note that this data set was not standardized for size effects because the calling individuals have not been collected following call record. In an analogous procedure, the release call data sets were treated, but adjusted for size (=SVL), whereas temperature was considered constant for all records. The procedure forward selection limited the number of call parameters in the final model to that subset, which maximized the rate of correct classification of calls. The classification functions compare the a priori call assignment to a species with that expected from the range of variation of the three taxon groups. If the scores suggest another taxon than the actual one, the call is considered a false assignment. The rates of false assignments of *P. ridibundus* calls to *P. bedriagae* and of *P. bedriagae* calls to *P. caralitanus* were used as a quantitative measure for the degree of distinctiveness.

Significance level was set at alpha = 0.05. All calculations were performed using the statistical package Statgraphics Centurion, version XVIII (Statpoint Inc., Warrenton, VA, USA, 2018).

## 3. Results

**Figure 1:**
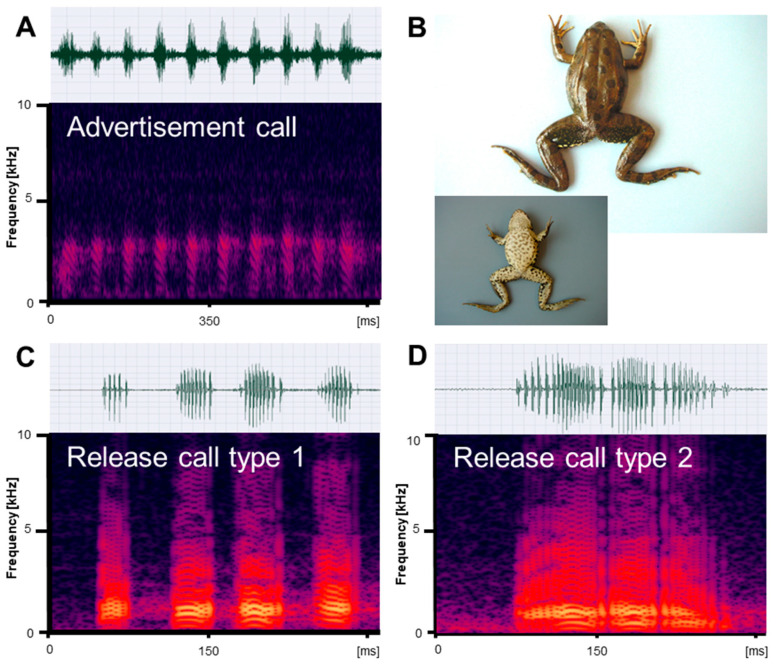
Mating vocalizations of *Pelophylax ridibundus* from Edirne. (**A**) Male advertisement call, recorded at 18.2 °C; (**B**) dorsal and ventral view of a male (SVL 66.5 mm); (**C**) release call type 1, recorded at 22 °C; (**D**) release call type 2, recorded at 21.5 °C. Calls are shown as audio spectrograms and oscillograms.

**Figure 2:**
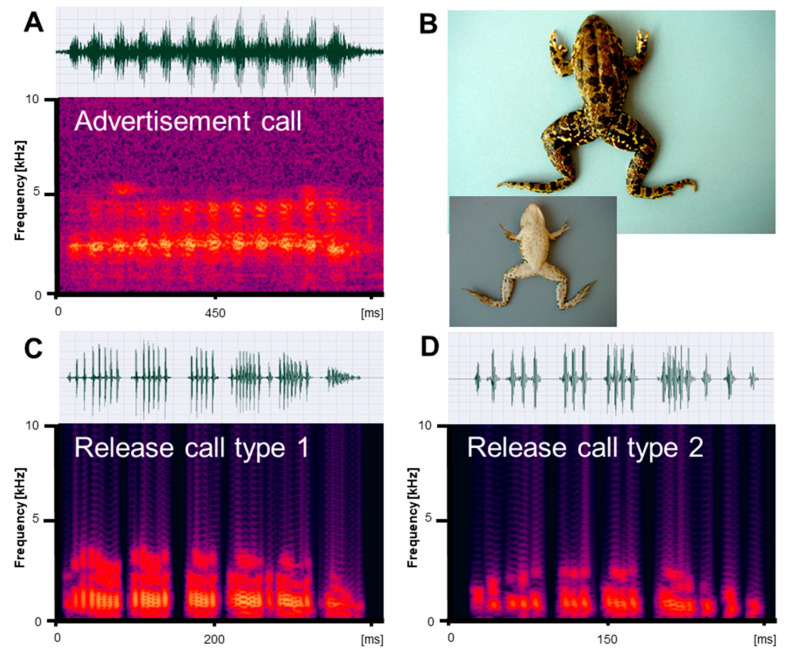
Mating vocalizations of *Pelophylax bedriagae* from Izmir. (**A**) Male advertisement call, recorded at 20 °C; (**B**) dorsal and ventral view of a male (SVL 74.4 mm); (**C**) release call type 1, recorded at 22 °C; (**D**) release call type 2, recorded at 22 °C. Calls are shown as audio spectrograms and oscillograms.

**Figure 3:**
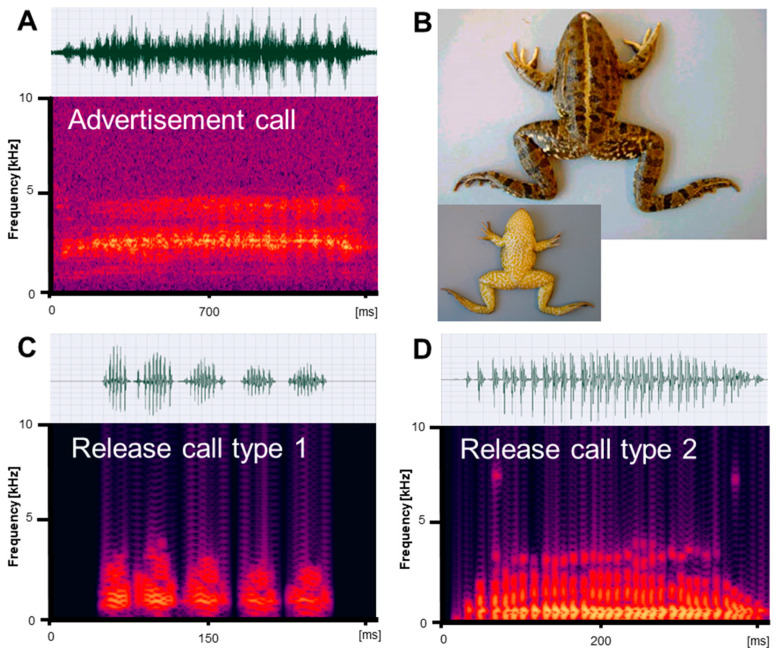
Mating vocalizations of *Pelophylax caralitanus* from Lake Beyşehir. (**A**) Male advertisement call, recorded at 20.8 °C; (**B**) dorsal and ventral view of a male (SVL 95.1 mm); (**C**) release call type 1, recorded at 21.5 °C; (**D**) release call type 2, recorded at 20 °C. Calls are shown as audio spectrograms and oscillograms.

Vocalizations recorded in reproductive *Pelophylax ridibundus, P. bedriagae,* and *P. caralitanus* during mating included three types of calls: (1) advertisement calls given exclusively by males to attract conspecific females within the natural habitat; (2) type 1 release calls given by males and females in response to artificial amplexus and consisting of several pulse groups; and (3) type 2 release calls given by males and females in response to artificial amplexus and consisting of a single pulse group (Figure 1, Figure 2 and Figure 3). In their natural environment, males also gave territorial calls, which were not studied in detail.

### 3.1. Comparison of Advertisement Calls

We obtained call series of 67 *P. ridibundus* individuals, those of 34 *P. bedriagae* specimens, and those of 12 *P. caralitanus* individuals recorded exclusively at Lake Beyşehir. The advertisement calls of the three water frog taxa shared the same basic temporal structure, i.e., each call consisted of a varying number of pulse groups spaced by regular inter-pulse group intervals (Figure 1A, Figure 2A and Figure 3A). Each pulse group consisted of pulses, which varied in number within a call. The dominant frequency was similar in all frogs and varied between 1800 and 2400 kHz (Table 1). Apart from the dominant frequency band, the audio spectrogram included lower and higher frequency bands with less energy.

**Figure 4:**
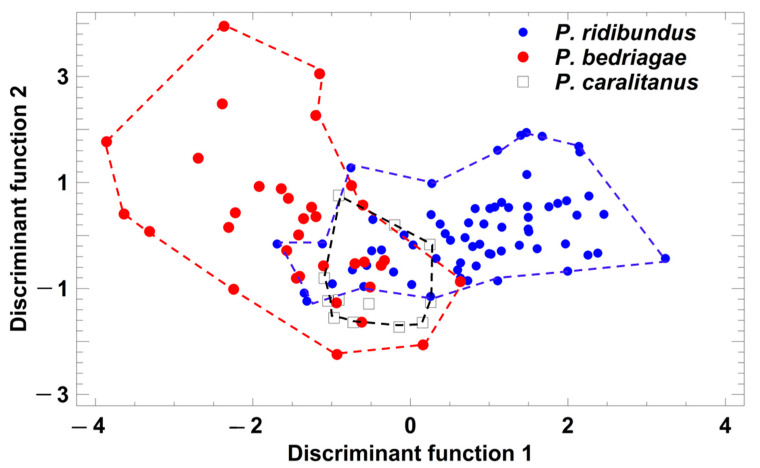
Grouping of *Pelophylax* taxa by a discriminant analysis based on the studentized residuals of advertisement call parameters that were regressed on ambient water temperature. Each symbol represents the call of one individual. For statistical details, see text.

Taxon-specific comparisons of the eight temperature-adjusted call parameters demonstrated that *P. ridibundus* differed significantly from the other taxa in six parameters (Table 1), and only call duration and dominant frequency did not vary among the taxa. In contrast, *P. bedriagae* and *P. caralitanus* differed significantly in only two parameters (pulse group duration, pulse repetition rate per pulse group; Table 1). A discriminant analysis (procedure: forward selection) yielded a model combining two parameters (studentized residuals of pulses per pulse group (sPPG) and of pulse repetition rate per pulse group (sPRPG)) in two significant discriminant functions: (1) DF1 = 1.236 × sPPG − 0.4184 × sPRPG; eigenvalue = 1.06, Χ^2^ = 91.7, df = 4, *p* < 0.0001; (2) DF2 = −0.574 × sPPG + 1.297 × sPRPG; eigenvalue = 0.12, Χ^2^ = 12.1, df = 1, *p* = 0.0005. The rate of correct call classification was 77.6% in *P. ridibundus*, 61.8% in *P. bedriagae*, and 75% in *P. caralitanus,* with all rates exceeding the rate of 33% for random allocation (Figure 4). The rate of confusion of *P. ridibundus* calls with *P. bedriagae* calls was 6.0%, and that of *P. bedriagae* calls with *P. caralitanus* calls was 35.3%. The scores of *P. caralitanus* overlapped completely with those of *P. bedriagae*.

### 3.2. Comparison of Release Calls

Submitted to artificial amplexus, two types of release calls were evoked: type 1, including several pulse groups, and type 2, consisting of a single pulse group with irregularly spaced pulses (Figure 1C,D, Figure 2C,D and Figure 3C,D). The response type and frequency differed among taxa: (1) seven *P. ridibundus* produced exclusively type 1 calls, two produced type 2 calls, and four did not respond; (2) two *P. bedriagae* gave type 1 calls (one switching to type 2 after some time), five exclusively gave type 2 calls, and five did not respond; (3) two *P. caralitanus* gave exclusively type 1 calls, fifteen gave type 2 calls, and seven did not respond. Independent of taxon, sex, and call type, the dominant frequency decreased gradually with increasing SVL, averaging about 1000 Hz in 62 mm individuals to about 600 Hz in a 121 mm female. The linear regression model (slope: −8.25 [Hz/mm SVL], intercept: 1488 [Hz]) explained 48.0% of frequency variation (r = −0.69; F_1,854_ = 625.1, *p* < 0.0001).

#### 3.2.1. Type 1 Calls

We obtained a total of 145 *P. ridibundus* calls, 29 *P. bedriagae* calls, and 24 *P. caralitanus* calls. The calls of the three water frog taxa shared the same basic temporal structure, resembling the advertisement call, i.e., each call consisted of several pulse groups with varying numbers of pulses (Figure 1C, Figure 2C and Figure 3C). The dominant frequency was considerably lower than in the advertisement calls and had several harmonics (Table 1 and Table 2).

**Figure 5:**
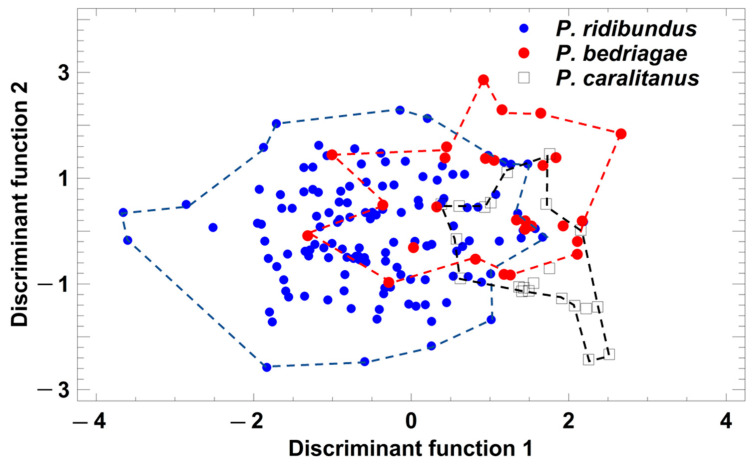
Grouping of *Pelophylax* taxa by a discriminant analysis based on the studentized residuals of female call parameters that were regressed on SVL. Each symbol represents the call of one individual. For statistical details, see text.

Taxon-specific comparisons of the eight SVL-adjusted call parameters showed that calls were considerably more similar than the corresponding advertisement (Table 2). Call duration, pulse group repetition rate, and pulses per call did differ significantly among taxa. *P. ridibundus* and *P. bedriagae* differed in three parameters and *P. bedriagae* and *P. caralitanus* in four (Table 2). A discriminant analysis (procedure: forward selection) yielded a model including five parameters (studentized residuals of call duration (sCD), pulse groups per call (sPG), dominant frequency (sDF), and pulse repetition rate per pulse group (sPRPG) and of pulses per pulse group (sPPG)) in two significant discriminant functions: (1) DF1 = −1.319 × sCD + 1.317 × sPG + 0.591 × sDF − 1.393 × sPRPG + 1.066 × sPPG; eigenvalue = 0.60, Χ^2^ = 98.8, df = 10, *p* < 0.0001; (2) DF2 = −0.201 × sCD + 0.920 × sPG + 0.079 × sDF + 0.054 × sPRPG − 0.276 × sPPG; eigenvalue = 0.10, Χ^2^ = 16.3, df = 4, *p* = 0.0026. The rate of correct classification was 72.5% in *P. ridibundus*, 48.2% in *P. bedriagae*, and 63.6% in *P. caralitanus* (Figure 5). The rate of erroneous classifications of *P. ridibundus* calls as *P. bedriagae* calls was 15.2%, and that of *P. bedriagae* calls as *P. caralitanus* calls was 36.4%. Besides a taxon-specific segregation range of discriminant scores, the three taxa showed a common range of overlap.

#### 3.2.2. Type 2 Calls

We obtained a total of 21 *P. ridibundus* calls, 184 *P. bedriagae* calls, and 458 *P. caralitanus* calls. The calls of the three water frog taxa shared the same basic temporal structure, i.e., each call consisted of a single pulse group with varying numbers of pulses (Figures Figure 1D, Figure 2D and Figure 3D). Often, the inter-pulse intervals varied within a call, maybe due to the omission of a pulse. The dominant frequency was considerably lower than in the advertisement calls, similar to that of release call type 1, and had several harmonics (Table 1 and Table 3).

**Figure 6:**
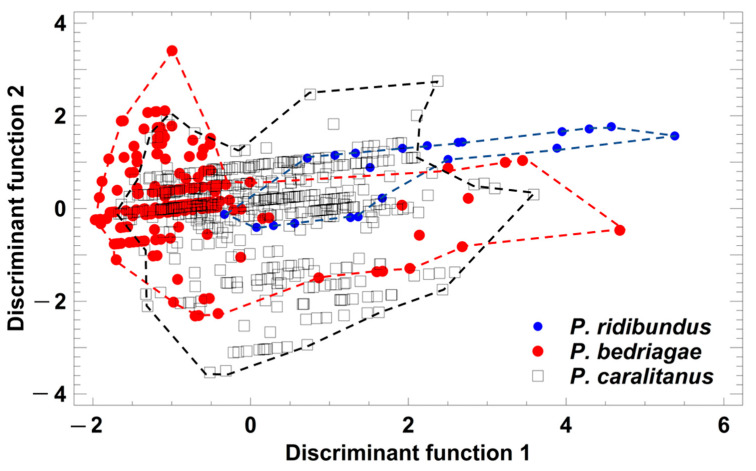
Grouping of *Pelophylax* taxa by a discriminant analysis based on the studentized residuals of release call parameters that were regressed on SVL. Each symbol represents the call of one individual. For statistical details, see text.

Taxon-specific comparisons of the five SVL-adjusted call parameters demonstrated that *P. ridibundus* release calls were significantly shorter and had more pulses than those of the other taxa (Table 3). *P. bedriagae* and *P. caralitanus* calls differed significantly in three parameters (pulses per call, pulse repetition rate, dominant frequency; Table 3). A discriminant analysis (procedure: forward selection) yielded a model including two parameters (studentized residuals of dominant frequency (sDF) and of pulse repetition rate (sPR)) in two significant discriminant functions: (1) DF1 = −0.134 × sDF + 0.986 × sPR; eigenvalue = 0.34, Χ^2^ = 218.6, df = 4, *p* < 0.0001; (2) DF2 = 0.992 × sDF + 0.174 × sPR; eigenvalue = 0.04, Χ^2^ = 25.4, df = 1, *p* < 0.0001. The rate of correct classification was 66.76% in *P. ridibundus*, 87.5% in *P. bedriagae*, and 50.8% in *P. caralitanus* (Figure 6). The rate of false classifications of *P. ridibundus* calls as *P. bedriagae* calls was 4.9%, and that of *P. bedriagae* calls as *P. caralitanus* calls was 33.9%. The taxon-specific range of discriminant scores showed the widest overlap of the vocalization types studied.

## 4. Discussion

We provide evidence that the differentiation of mating vocalizations in the Turkish water frog taxa mirrors exactly the genetic differentiation found in Cyt b, 12S rRNA, and 16S rRNA loci [16]. This statement is derived from a two-step process consisting first of the identification of the function of the calls we have recorded, i.e., the behavioral context in which the calls are given during mating. The second step is an evaluation of the call structure variation as a means of premating isolation. Finally, we integrate all available information to answer the question of whether *P. bedriagae* and *P. caralitanus* are distinct species or not.

### 4.1. Validation of Call Identification

Structural features of advertisement calls given by water frogs in Turkish Thrace coincide with the variation range of those recorded in topotypical *P. ridibundus* (Atyrau, Kazakhstan) and those in the neighboring Greek Thrace [13,40]. There is no doubt about the species assignment of these frogs to *P. ridibundus*. The advertisement calls of water frogs at the Aegean coast in Izmir agree with the features of those recorded in topotypical *P. bedriagae* (Damascus, Syria) and those in Israel and Iran, so that species identification as *P. bedriagae* is corroborated as well [41,42,43]. As for the Beyşehir taxon, to date, the only published advertisement calls are recordings by Hans Schneider [14]. We did not observe character displacement of call parameters in the *bedriagae*/*caralitanus* pair.

The calls of type 1 given in artificial amplexus agree in temporal structure and spectral features with the release calls previously recorded in *P. ridibundus* at several localities within the geographical range [44,45,46,47]. Given the structural similarity to the corresponding advertisement call of each taxon, but at a considerably lower dominant frequency, we suggest that this signal is directed to the acoustic communication with conspecifics. In *P. bedriagae,* a specific study on release vocalizations is not available. Accidental recordings of release call type 2 were obtained in Syria and Israel by Schneider, who misinterpreted this call type as territorial call 3 [41,42]. As early as in 1969, the same call type was recorded in *P. esculentus* in response to artificial amplexus, and later in diploid males from the Ukraine as well [47,48]. The similarity of this release call type in many water frog taxa suggests that it might function as a signal for interspecific communication, as several taxa often occur syntopically. The presence of two types of release calls (one directed to conspecifics, one to heterospecifics) is not unusual in anurans, as demonstrated in neotropical toads of the genus *Rhinella* as well [37,38].

### 4.2. Acoustic Premating Barriers and Taxon Delimitation

The current genetic baseline for taxon delimitation of water frogs in Turkey is the most extensive molecular study on the genetic differentiation by Bülbül et al. [16]. They found a deep lineage splitting between *P. ridibundus* and *P. bedriagae*/*P. caralitanus* at a genetic distance of 3.1% and a low genetic variation among populations of the latter taxon pair at 0.6–1.2% uncorrected P-distance. The differentiation of the *P. ridibundus* lineage is clearly at the species level, and the *P. bedriagae* and *P. caralitanus* lineages are certainly below, as concluded by Bülbül et al. [16]. Still, two populations referred to as *P. caralitanus* were assigned to a well-supported distinct subclade, indicating a speciation process in progress within the complete *P. bedriagae* clade.

Our approach focusing on the potential premating isolation among the water frog taxa in Turkey by mating vocalizations mirrors the molecular view. As predicted, and in agreement with an earlier study [14], the rate of false classification of *P. ridibundus* advertisement calls as those of *P. bedriagae* was only at 6%, whereas that of *P. bedriagae* and *P. caralitanus* calls was at 35%, indicating an ineffective acoustic premating isolation. The leaky isolation from coastal *P. bedriagae* populations is also apparent from karyological data and introgression [27,30]. As predicted, the release call structure was more similar than that of the advertisement calls, and in type 2 this was stronger than in type 1. Evidently, overall bioacoustics similarity in the *P. bedriagae*/*P. caralitanus* taxon pair exceeded considerably that of the *P. ridibundus/P. bedriagae* species pair, indicating a deeper lineage splitting. Integrating variation of advertisement and release call structure leaves no doubt that the mating vocalizations within the taxon *P. bedriagae*/*P. caralitanus* are too similar to constitute effective premating barriers and to prevent significant gene flux.

## 5. Conclusions

Our quantitative assessment of the effectiveness of acoustic premating isolation in agreement with available genetic, karyological, and morphological data provides evidence that the Beyşehir frogs do not deserve species status and that *P. caralitanus* should be considered a junior synonym of *P. bedriagae*. We acknowledge that the Lake District water frogs differ in some respects from the nominal *P. bedriagae* populations, indicating an ongoing speciation process or the remixing of temporarily isolated populations. Therefore, we conclude that the *caralitanus* taxon forms an integral part of the *P. bedriagae* superspecies. We agree with Arikan and Bülbül et al. [16,19] that the subspecies level (=*P. bedriagae caralitanus*) may indicate the current state of the speciation process if one supports taxonomic levels below a species (for a critical discussion of this issue, see [49]). From an evolutionary point of view, we predict that an insignificant gene flow will lead to a speciation in the future, whereas a moderate to high gene flow will complete homogenization with *P. bedriagae*. Regardless, the data base “Amphibians of the World” should recognize the overwhelming evidence that the assumed species status was based on flawed analyses of morphometric data and a claimed bioacoustic differentiation that has never been evidenced in a published paper [17,28,31].

The consequence for the diversity of anuran amphibians in Turkey and resulting conservation management is significant: instead of 16 species, there are only 15, and more importantly, the number of endemic anurans shrinks to only one (*Rana tavasensis*) [2,4,17]. The prognosed reduction of the distribution range of the *caralitanus* taxon during the next five decades due to climate change is still alarming [50]. The consequence may be a loss of the current genetic diversity within the *P. bedriagae* superspecies, either by extinction of peculiar lineages or by an increased homogenization of the gene pool.

## Figures and Tables

**Table 1:** Temperature-adjusted advertisement call parameters measured in *Pelophylax* spp. Data are given as untransformed, temperature-adjusted arithmetic means and corresponding standard error. For statistical comparisons among taxa, we used log10-normalized data with ambient air temperature as a co-variable in an ANCOVA. Hyphenated letters indicate homogeneous groups that differ at *p* < 0.05.

Call Parameter	*P. ridibundus*	*P. bedriagae*	*P. caralitanus*	Significance
Call duration [ms]	609 ± 17	625 ± 24	680 ± 41	*p* = 0.2734
Pulse groups per call [N]	7.0 ± 0.2 ^a^	8.1 ± 0.3 ^b^	8.5 ± 0.4 ^b^	*p* = 0.0003
Pulse group duration [ms]	58.8 ± 0.9 ^a^	44.1 ± 1.2 ^c^	53.0 ± 2.1 ^b^	*p* < 0.0001
Pulse group interval [ms]	38.6 ± 1.1 ^a^	45.1 ± 1.1 ^b^	41.2 ± 1.7 ^b^	*p* = 0.0070
Pulse group repetition rate [N/s]	11.6 ± 0.2 ^a^	13.1 ± 0.3 ^b^	12.8 ± 2.7 ^b^	*p* = 0.0002
Pulses per pulse group [N]	20.1 ± 0.3 ^a^	12.2 ± 0.5 ^b^	13.0 ± 0.8 ^b^	*p* < 0.0001
Pulse repetition rate per pulse group [N/s]	343 ± 7 ^a^	287 ± 9 ^b^	244 ± 16 ^c^	*p* < 0.0001
Dominant frequency [Hz]	2209 ± 51	2183 ± 61	1983 ± 92	*p* = 0.4632

**Table 2:** Release call type 1 parameters measured in *Pelophylax* spp. Data are given as arithmetic means and corresponding standard error. Statistical comparisons among taxa were based on log10-normalized data using SVL as co-variable in an ANCOVA. Hyphenated letters indicate homogeneous groups that differ at *p* < 0.05.

Call Parameter	*P. ridibundus*	*P. bedriagae*	*P. caralitanus*	Significance
Call duration [ms]	248 ± 7	254 ± 16	213 ± 19	*p* = 0.5727
Pulse groups per call [N]	3.8 ± 0.1 ^a^	4.4 ± 0.2 ^b^	3.2 ± 0.2 ^a^	*p* = 0.0005
Pulse group duration [ms]	45.3 ± 1.0 ^b^	40.2 ± 2.2 ^a^	46.8 ± 2.8 ^b^	*p* = 0.0148
Pulse group interval [ms]	25.9 ± 0.9 ^a^	21.6 ± 1.9 ^a^	28.7 ± 2.5 ^b^	*p* = 0.0041
Pulse group repetition rate [N/s]	16.9 ± 0.4	18.6 ± 0.9	11.4 ± 1.1	*p* = 0.0746
Pulses per call [N]	7.5 ± 0.1	6.8 ± 0.3	7.4 ± 0.4	*p* = 0.0504
Pulse repetition rate per pulse group [N/s]	183.5 ± 3.0 ^b^	178.0 ± 6.7 ^b^	145.8 ± 8.5 ^a^	*p* = 0.0185
Dominant frequency [Hz]	895 ± 10 ^a^	978 ± 21 ^b^	998 ± 27 ^b^	*p* < 0.0001

**Table 3:** Release call type 2 parameters measured in *Pelophylax* spp. Data were pooled for males and females and are given in arithmetic means and corresponding standard error. Statistical comparisons among taxa were based on log10-normalized data using SVL as co-variable in an ANCOVA. Hyphenated letters indicate homogeneous groups that differ at *p* < 0.05.

Call Parameter	*P. ridibundus*	*P. bedriagae*	*P. caralitanus*	Significance
Call duration [ms]	214 ± 12 ^a^	247 ± 5 ^b^	243 ± 3 ^b^	*p* = 0.0040
Pulse groups per call [N]	1	1	1	-
Pulses per call [N]	23.6 ± 1.3 ^b^	13.0 ± 0.5 ^a^	19.0 ± 0.3 ^b^	*p* < 0.0001
Pulse repetition rate [N/s]	120.0 ± 5.1 ^c^	52.7 ± 1.9 ^a^	82.7 ± 1.2 ^b^	*p* < 0.0001
Dominant frequency [Hz]	867 ± 32 ^a^	842 ± 12 ^a^	742 ± 2 ^b^	*p* < 0.0001

## Data Availability

Call data are available on request from the corresponding author (US).

## References

[1] Maiorano L., Amori G., Capula M., Falcucci A., Masi M., Montemaggiori A., Pottier J., Psomas A., Rondinini C., Russo D. (2013). Threats from Climate Change to Terrestrial Vertebrate Hotspots in Europe. PLoS ONE.

[2] Karataş A., Filiz H., Erciyas-Yavuz K., Özeren S.C., Tok C.V., Öztürk M. (2021). The vertebrate biodiversity of Turkey. Biodiversity, Conservation and Sustainability in Asia: Volume 1: Prospects and Challenges in West Asia and Caucasus.

[3] Eken G., Isfendiyaroğlu S., Yeniyurt C., Erkol I.L., Karataş A., Ataol M. (2016). Identifying key biodiversity areas in Turkey: A multi-taxon approach. Int. J. Biodivers. Sci. Ecosyst. Serv. Manag..

[4] Yaşar Ç., Çiçek K., Mulder J., Tok C. (2021). The distribution and biogeography of amphibians and reptiles in Turkey. North-West. J. Zool..

[5] Başkale E., Kaya U. (2009). Richness and Distribution of Amphibian Species in Relation to Ecological Variables in Western Aegean Region of Turkey. Ekoloji.

[6] Ambarlı D., Zeydanlı U.S., Balkız Ö., Aslan S., Karaçetin E., Sözen M., Ilgaz Ç., Gürsoy Ergen A., Lise Y., Demirbaş Çağlayan S. (2016). An overview of biodiversity and conservation status of steppes of the Anatolian Biogeographical Region. Biodivers. Conserv..

[7] Erismis U.C., Konuk M., Yoldas T., Agyar P., Yumuk D., Korcan S.E. (2014). Survey of Turkey’s endemic amphibians for chytrid fungus *Batrachochytrium dendrobatidis*. Dis. Aquat. Org..

[8] Çiçek K., Ayaz D., Afsar M., Bayrakci Y., Peksen C.A., Cumhuriyet O., Ismail I.B., Yenmis M., Ustundag E., Tok C.V. (2021). Unsustainable harvest of water frogs in southern Turkey for the European market. Oryx.

[9] Başkale E., Kaya U. (2012). Decline of the Levantine Frog, *Pelophylax bedriagae* Camerano, 1882, in the western Aegean Region of Turkey: Changes in population size and implications for conservation (Amphibia: Ranidae). Zool. Middle East.

[10] Bodenheimer F.S. (1944). Introduction into the knowledge of the Amphibia and Reptilia of Turkey. Rev. Fac. Sci. Univ. Istanb..

[11] Basoglu M., Özeti N. (1973). Türkiye Amfibileri.

[12] Schneider H., Sinsch U., Nevo E. (1992). The lake frogs in Israel represent a new species. Zool. Anz..

[13] Schneider H., Sinsch U. (1992). Mating call variation in lake frogs referred to as *Rana ridibunda* Pallas, 1771. Taxonomic implications. Z. Für Zool. Syst. Und Evol..

[14] Schneider H., Sinsch U. (1999). Taxonomic reassessment of Middle Eastern water frogs: Bioacoustic variation among populations considered as *Rana ridibunda*, *R. bedriagae* or *R. levantina*. J. Zool. Syst. Evol. Res..

[15] Berger L., Uzzell T., Hotz H.J. (1994). Postzygotic reproductive isolation between Mendelian species of European water frogs. Zool. Pol..

[16] Bülbül U., Matsui M., Kutrup B., Eto K. (2011). Taxonomic Relationships among Turkish Water Frogs as Revealed by Phylogenetic Analyses using mtDNA Gene Sequences. Zool. Sci..

[17] Frost D.R. (2023). Amphibian Species of the World: An Online Reference.

[18] Sinsch U., Schneider H. (1999). Taxonomic reassessment of Middle Eastern water frogs: Morphological variation among populations considered as *Rana ridibunda*, *R. bedriagae* or *R. levantina*. J. Zool. Syst. Evol. Res..

[19] Arikan H. (1988). On a new form of *Rana ridibunda* (Anura, Ranidae) from Turkey. Istanb. Üniv. Fen Fak. Biyol. Der..

[20] Atatür K.M., Arikan H., Mermer A. (1990). A taxonomical investigation on *Rana ridibunda* Palla (Anura, Ranidae) populations from the Lakes District-Anatolia. Istanb. Üniversitesi Fen. Fakültesi Biyol. Derg..

[21] Alpagut N., Falakali B. (1995). Karyotype analysis of two *Rana ridibunda* (Ranidae: Anura) populations in Turkey. Isr. J. Zool..

[22] Budak A., Tok C.V., Ayaz D. (2000). On specimens of *Rana ridibunda* Pallas, 1771 (Anura: Ranidae) collected from Isikli Lake (Civril-Denizli). Turk. J. Zool..

[23] Kaya U., Cevik E., Erismis U.C. (2002). New distributional records for *Rana bedriagae caralitana* in Anatolia. Turk. J. Zool..

[24] Tosunoğlu M., Ayaz D., Göçmen B. (2005). On Specimens of *Rana ridibunda* Pallas, 1771 (Anura: Ranidae) Collected from Yağmapınar (Karapinar-Konya). Anadolu Üniversitesi Bilim Ve Teknol. Derg..

[25] Ayaz D., Tok C.V., Mermer A., Tosunoğlu M., Afsar M., Çiçek K. (2006). A New Locality for *Rana ridibunda caralitana* Arıkan, 1988 (Anura: Ranidae) in the Central Anatolia. Su Ürünleri Derg..

[26] Akin C., Bilgin M., Bilgin C.C. (2010). Discordance between ventral colour and mtDNA haplotype in the water frog *Rana (ridibunda) caralitana*, 1988 Arikan. Amphib.-Reptil..

[27] Akin C., Bilgin C.C., Beerli P., Westaway R., Ohst T., Litvinchuk S.N., Uzzell T., Bilgin M., Hotz H., Guex G.-D. (2010). Phylogeographic patterns of genetic diversity in eastern Mediterranean water frogs were determined by geological processes and climate change in the Late Cenozoic. J. Biogeogr..

[28] Jdeidi T., Bilgin C.C., Kence M. (2001). New localities extend the range of *Rana bedriagae caralitana* Arikan, 1988 (Anura: Ranidae) further west and suggest specific status. Turk. J. Zool..

[29] Plötner J., Ohst T., Böhme W., Schreiber R. (2001). Divergence in mitochondrial DNA of Near Eastern water frogs with special reference to the systematic status of Cypriote and Anatolian populations (Anura, Ranidae). Amphib.-Reptil..

[30] Alpagut-Keskin N., Falakali-Mutaf B. (2006). Rod-Shaped Bivalents indicate new assemblage among Anatolian Water Frog Populations. Amphib.-Reptil..

[31] Jdeidi T., Bilgin C.C., Kence M. Morphometric and bioacoustic studies in the Water Frog (*Rana ridibunda*) complex in Turkey. Proceedings of the Sixth International Congress of Vertebrate Morphology.

[32] Schneider H., Sinsch U., Sofianidou T. (1993). The water frogs of Greece: Bioacoustic evidence for a new species. Z. Für Zool. Syst. Und Evol..

[33] Twomey E., Mayer M., Summers K. (2015). Intraspecific Call Variation in the Mimic Poison Frog Ranitomeya imitator. Herpetologica.

[34] Stewart K.A., Austin J.D., Zamudio K.R., Lougheed S.C. (2016). Contact zone dynamics during early stages of speciation in a chorus frog (*Pseudacris crucifer*). Heredity.

[35] Weaver S.J., Callaghan C.T., Rowley J.J.L. (2020). Anuran accents: Continental-scale citizen science data reveal spatial and temporal patterns of call variability. Ecol. Evol..

[36] Leary C.J. (1999). Comparison between Release Vocalizations Emitted during Artificial and Conspecific Amplexus in *Bufo americanus*. Copeia.

[37] Di Tada I.E., Martino A., Sinsch U. (2001). Release vocalizations in neotropical toads (*Bufo*): Ecological constraints and phylogenetic implications. J. Zool. Syst. Evol. Res..

[38] Di Tada I.E., Sinsch U. (2023). Geographical variation of advertisement and release calls in toads referred to as *Rhinella diptycha* (Anura: Bufonidae). Salamandra.

[39] Köhler J., Jansen M., Rodríguez A., Kok P.J.R., Toledo L.F., Emmrich M., Glaw F., Haddad C.F.B., Rödel M.-O., Vences M. (2017). The use of bioacoustics in anuran taxonomy: Theory, terminology, methods and recommendations for best practice. Zootaxa.

[40] Schneider H., Egiasarjan E. (1991). The structure of the calls of lake frogs (*Rana ridibunda*: Amphibia) in the Terra Typica Restricta. Zool. Anz..

[41] Schneider H. (1997). Calls and reproductive behaviour of the water frogs of Damascus, Syria (Amphibia: Anura: *Rana bedriagae* Camerano, 1882). Zool. Middle East.

[42] Schneider H. (1999). Calls of the Levantine frog, *Rana bedriagae*, at Birket Ata, Israel (Amphibia: Anura). Zool. Middle East.

[43] Pesarakloo A., Najibzadeh M., Rastegar-Pouyani N., Rastegar-Pouyani E. (2018). Taxonomic survey of water frog populations of *Pelophylax bedriagae* (Anura: Ranidae) in western Iran: A morphometric and bioacoustic approach. Biologia.

[44] Schneider H., Brzoska J. (1981). Die Befreiungsrufe der mitteleuropäischen Wasserfrösche. Zool. Anz..

[45] Zamfirescu Ş. (2002). Comparison between water frogs (*Rana esculenta* complex) release calls. An. Ştiinţifice Ale Univ. “Alexandru Ioan Cuza” Din Iaşi S. Biol. Anim..

[46] Chernyshov K. Variability of advertisement calls and release calls of green frogs in the Moscow oblast, Russia. Proceedings of the Herpetologia Petropolitana–12th Ordinary General Meeting of the Societas Europaea Herpetologica.

[47] Fedorova A., Shabanov D. (2023). Differences in release calls of the hybrid water frog *Pelophylax esculentus* and its parental species *Pelophylax ridibundus* (Anura: Ranidae) in Ukraine. Biologia.

[48] Wahl M. (1969). Untersuchungen zur Bio-Akustik des Wasserfrosches *Rana esculenta* (L.). Oecologia.

[49] Burbrink F.T., Crother B.I., Murray C.M., Smith B.T., Ruane S., Myers E.A., Pyron R.A. (2022). Empirical and philosophical problems with the subspecies rank. Ecol. Evol..

[50] Kıraç A., Gidiş M., Mert A., Başkale E. (2022). Climate change and the fate of endemic Beysehir Frog, *Pelophylax caralitanus*. Amphib. Reptile Conserv..

